# Current Trends in Anterior Cruciate Ligament Reconstruction: A Review

**DOI:** 10.7759/cureus.378

**Published:** 2015-11-13

**Authors:** Raju Vaishya, Amit Kumar Agarwal, Sachin Ingole, Vipul Vijay

**Affiliations:** 1 Orthopaedics, Indraprastha Apollo Hospitals

**Keywords:** anterior cruciate ligament reconstruction, grafts, fixation devices, hamstring, rehabilitation

## Abstract

Anterior cruciate ligament reconstruction (ACLR) is an accepted and established surgical technique for anterior cruciate ligament (ACL) injuries and is now being practiced across the globe in increasing numbers. Although most patients get good to excellent results in the short-term after ACLR, its consequences in the long-term in prevention or acceleration of knee osteoarthritis (OA) are not yet well-defined. Still, there are many debatable issues related to ACLR, such as the appropriate timing of surgery, graft selection, fixation methods of the graft, operative techniques, rehabilitation after surgery, and healing augmentation techniques. Most surgeons prefer not to wait long after an ACL injury to do an ACLR, as delayed reconstruction is associated with secondary damages to the intra- and periarticular structures of the knee. Autografts are the preferred choice of graft in primary ACLR, and hamstring tendons are the most popular amongst surgeons. Single bundle ACLR is being practiced by the majority, but double bundle ACLR is getting popular due to its theoretical advantage of providing more anatomical reconstruction. A preferred construct is the interference fixation (Bio-screw) at the tibial site and the suspensory method of fixation at the femoral site. In a single bundle hamstring graft, a transportal approach for creating a femoral tunnel has recently become more popular than the trans-tibial technique. Various healing augmentation techniques, including the platelet rich plasma (PRP), have been tried after ACLR, but there is still no conclusive proof of their efficacy. Accelerated rehabilitation is seemingly more accepted immediately after ACLR.

## Introduction and background

The anterior cruciate ligament (ACL) is one of the most commonly injured ligaments of the knee in contact sports players. It accounts for about 200,000 injuries per year in the United States alone [1]. With the same activity level, females are four times more prone to ACL injuries than males [2]. There are various predisposing factors for ACL injury, which includes neuromuscular and biomechanical abnormalities, mutations within collagen producing genes like the COL5A1 and COL1A1 genes, female sex hormones, abnormal joint laxity [3], and primary structural influences of the knee [4-5].

Conservative management is useful in sedentary patients, but for other physically active patients, it is associated with a significant drawback when they can't resume high-level sports activities successfully [6]. Moreover, chronic ACL insufficiency may be associated with subsequent meniscal and articular cartilage injury and residual knee instability [7]. Hence, ACLR is now considered the treatment of choice in the majority of cases. Each year, ACLR accounts for 100,000 surgeries in the USA itself [8]. Arthroscopic ACLR is universally accepted as a highly successful operation with consistent results in the majority of the patients. The techniques of ACLR have seen an ocean of change in the past couple of decades in the areas of surgical techniques and understanding of the biomechanics.

This article reviews the available literature related to the timing of ACLR surgery, graft selection, fixation methods, fixation devices, healing augmentation, postoperative rehabilitation, and association with knee osteoarthritis (OA).

## Review

### Timing of surgery

Optimal timing of ACLR is a debatable issue, as both early and delayed ACLR are reported to be associated with adverse clinical outcomes. The timing of the surgery is influenced by various clinical and social factors. The clinical decision making depends upon preoperative swelling, edema, local temperature, and range of motion (ROM). There are various other social factors like surgeon’s preference, family, personal, or social obligations, as well as the mental preparation of the patient.

Arthrofibrosis is considered as one of the most common complications following early ACLR (Table 1).

**Table 1:** Complications related to the timing of ACLR

Time to Surgery	Early (< 3 weeks)	Late (> 1 year)
Complications and drawbacks	Arthrofibrosis Prolonged rehabilitation	Osteoarthritis Meniscal injury Osteochondral damage Ligament tear

The chances of arthrofibrosis increases if ACLR surgery is performed within three weeks after injury [9-11] compared to a delayed ACLR. Objective criteria, including preoperative swelling, edema, raised local temperature, and ROM, are important indicators for deciding the timing of surgery [12]. Bone edema also affects the purchase of interference screw. Eitzen, et al. [13] found that preoperative quadriceps strength influences clinical outcomes following reconstruction surgery as the patients with less than 80% quadriceps strength of the involved limb is associated with inferior outcomes at two years postoperatively. Hence, the author suggested that at least 80% of the original quadriceps muscle strength should be achieved preoperatively.

Some studies have favored delayed (three to 52 weeks) ACLR. Shelbourne, et al., in their study of 169 young athletes, found that the patients with ACLR performed within seven days of injury are more prone to develop arthrofibrosis than the patients in whom surgery is delayed for three weeks or more. Their study also confirmed that in the group who had an ACLR between one to three weeks after injury and adopted an accelerated rehabilitation program had fewer chances of arthrofibrosis as compared to the conventional group. Almekinders, et al. reported a decrease in early ROM in patients with ACLR done within a month's time but no long-term difference with ROM when compared to patients whose surgery was done longer than one month after injury. They also found that prolonged surgeries are associated with a limited early ROM. Passler, et al. found a significant increase in the incidence of arthrofibrosis in early (seven days) versus delayed (four weeks) group (17.6% versus 6.1%, respectively). Mayr, et al. documented that preoperative knee irritability (i.e., edema, swelling, the local rise of temperature, ROM, and pain) decides the outcome and incidence of arthrofibrosis after ACLR.

In general, an early ACLR is now considered better and gives excellent clinical outcomes. Delayed surgery for more than a year may be associated with an increased risk of secondary injuries to the menisci, articular cartilage, and collateral ligaments and, thus, leading to an early OA (Table 1). Smith, et al. [14] in their meta-analysis found the similar clinical outcome in patients undergoing ACLR within either three weeks or after six weeks of injury. Church and Keating [15], Kennedy, et al. [16], and Razi, et al. [17] reported a significant increase in meniscal tears and knee degenerative changes in patients in whom ACLR was done after one year of injury. Hence, according to them, ACLR should be done within 12 months of injury to avoid further meniscal and cartilage damage. Granan, et al. [18] reported a 1% increase in an odds ratio (for articular cartilage lesions) per month for every month from injury to surgery. Demirag, et al. [19] found that the incidence of meniscal and osteochondral lesions increases with a delay in ACLR, and it negatively affects the surgical outcome. We believe that an early ACLR (within three weeks of injury) may be associated with an increased risk of arthrofibrosis, but if done, then it must be supported by an accelerated rehabilitation program. Delayed surgery beyond one year is associated with significantly increased chances of secondary damage to the other intra-articular structures of the knee and may lead to early OA.

### Graft selection

**Figure 1:**
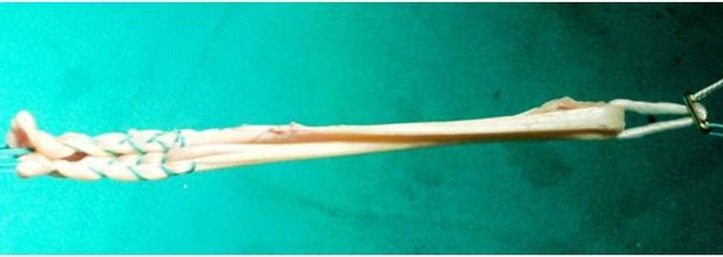
Quadrupled hamstring graft with an Endobutton

The choice of grafts in ACLR is usually surgeon-dependent, but may also be influenced by the availability of grafts and the patients’ choice. The most commonly used grafts include a) autografts, e.g., bone-patellar-tendon-bone (BPTB) and hamstring grafts (HS) (Figure 1), b) allografts, e.g., tibialis posterior tendon, Achilles tendon, tibialis anterior tendon, BPTB, and peroneus longus tendon, and c) synthetic grafts, e.g., polyester, carbon composites, etc. 

Romanini, et al. [20] recommended autografts over allografts in ACLR. The author also suggested considering allograft/artificial grafts in selected cases only. Maletis, et al. [21] found that allografts had a 3.02 times higher risk of aseptic revision than BPTB autografts. Hamstring tendon autografts had a 1.82 times greater likelihood of revision compared with BPTB autografts. They further commented that with increasing age, the risk of revision surgery decreases by 7%. They also observed that in females there are 2.26 times higher risk of revision surgery with the use of hamstring autograft compared to BTPB autograft. The risk factor for early revision in ACLR includes allograft use, hamstring tendon graft, and younger age.

It is still not clear which is the “best” autograft to use in ACLR. In a study, Shaerf, et al. [22] have documented the pros and cons of various commonly used grafts. A BPTB graft is associated with donor site morbidity but appears to have good graft stability and a return to high-level sports. An HS graft is an attractive, good all-around graft choice with easier harvesting, fewer donor site complications, and good results. Both sources of autograft are readily available in most patients and costs nothing extra, but do have some technical demands for safe and efficient harvest. Allograft has slightly poorer results in terms of re-rupture rates, but is valuable in situations where the availability of an autograft is a concern, such as in multi-ligament deficiencies or in the revision scenario. Allografts are expensive, but save time and undoubtedly remove one of the more technically demanding stages of ACLR (Table 2).

**Table 2:** Pros and cons of various grafts used for ACL reconstruction

Graft	Pros	Cons
Autograft	Readily available No disease transmission No sterilization hence good long term strength Readily accepted by body.	Increased surgical time Donor site morbidity
Allograft	No donor site morbidity Reduces surgical time Smaller incisions Availability of large graft without weakening of extensor or flexor apparatuses Useful in revision surgery	Availability Graft cost Disease transmission particularly HIV, Hepatitis Poor graft strength due to sterilization process Delayed graft incorporation
Synthetic graft	No donor site morbidity Reduces surgical time No disease transmission useful in revision surgery	Expensive Higher rate of graft failure Late inflammation

Allografts eliminate the potential for donor site morbidity but do not permit a faster return to sports. Synthetic grafts are slowly regaining popularity as these, too, show good results with no donor site morbidity and the ability to perform multi-ligament reconstructions without compromising the patella or hamstrings. They shorten operative time; however, the surgery is technically different. The concern about the safety of synthetic grafts needs to be confirmed, as they have been reported to be associated with high costs, increased graft failures, and synovitis in some cases.

The choice of autograft is also influenced by the concerns of longevity and their capability of providing long-term stability of these grafts.  Goddard, et al. [23] in a case-control study reported a 15-year graft survival rate of 83% (1.1% failure/year) in isolated ACL anatomical reconstruction using a hamstring tendon autograft. Paterno, et al. [24] reported an increase in anteroposterior knee laxity following ACLR in females as compared to males when hamstring autografts were used. However, there were no sex-based differences in AP laxity when BPTB graft was used. Therefore, ACLR with BPTB graft remains the gold standard as far as mechanical stability is concerned, irrespective of the sex of the patient.

Autografts are preferred over allografts as they are readily available and not as costly as an allograft. Hamstring autografts are the graft of choice, but they are associated with high revision rates and increased AP laxity postoperatively, especially in females as compared with BPTB grafts. Synthetic grafts are not used routinely but to be reserved for special situations like revision surgery. Thus, graft choice is to be done with the patient’s education and surgeon’s experience.

### Fixation devices

**Figure 2:**
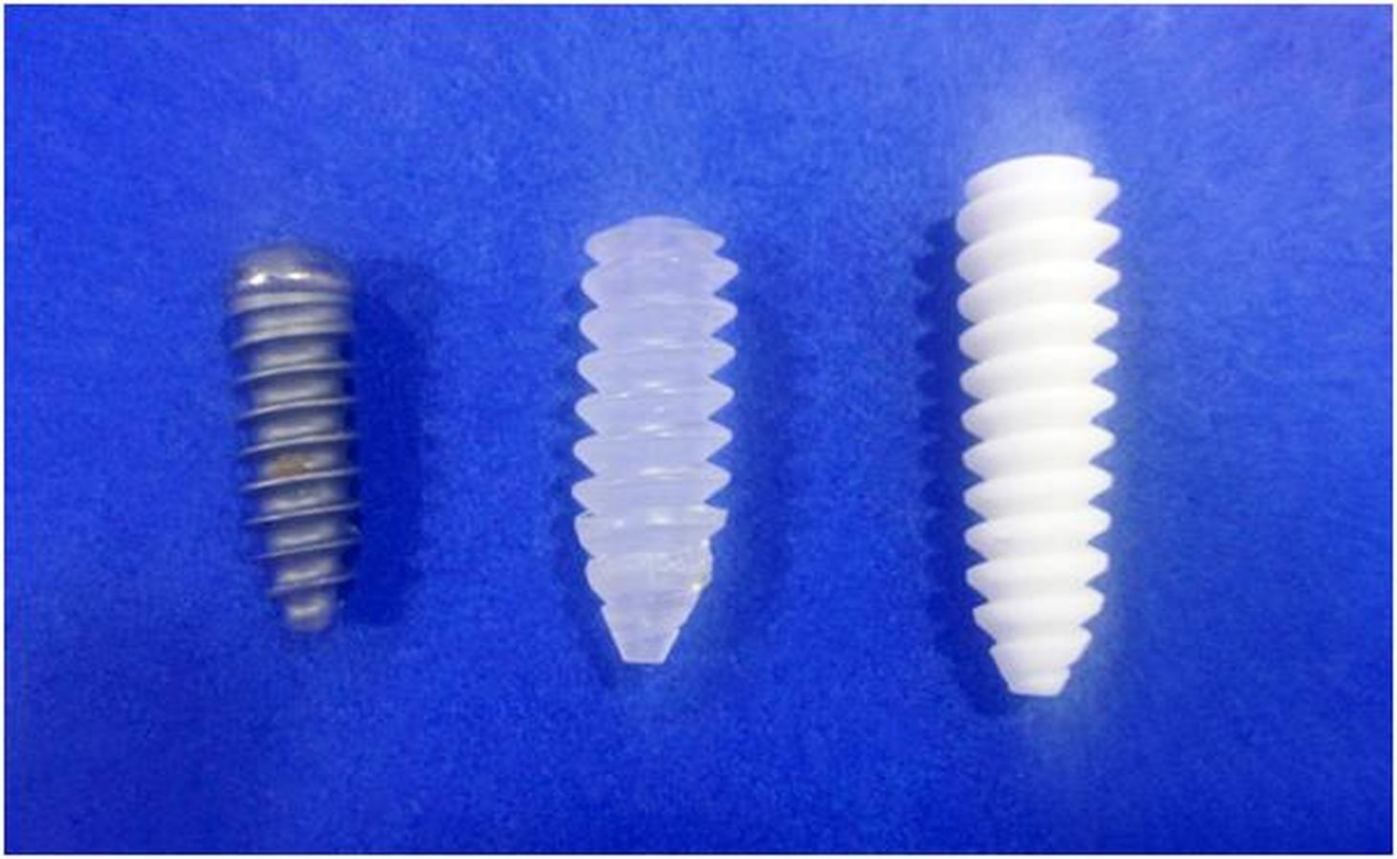
Various types of interference screws used for ACLR (Titanium, Bio, HA-coated: Right to left)

There are mainly two types of fixation devices used in ACLR in bone tunnels: A) Aperture fixation means the fixation of a graft at the opening of the bone tunnel like interference screws (Figure 2), Intrafix, etc., and B) suspensory fixation means fixation of the graft that is remote from the intra-articular space.

Aperture graft fixation device includes a screw, post, and washer, etc., whereas suspensory fixation of a graft is done using sutures suspended from a femoral fixation device like an EndoButton® (Smith & Nephew, Inc. Andover, MA, USA) or TransFix® (Arthrex inc,Naples, Florida,USA). The main purpose of these devices is to provide a secure fixation so that the graft gets proper healing into the tunnel. This further helps in starting early range of motion exercises and weight-bearing and, hence, the early return to sports without any loss of fixation strength (Table 3).

**Table 3:** Role of fixation devices

Fixation devices in ACLR:
Provide secure fixation of the graft
Allow graft healing within the tunnel
Allow immediate ROM and weight bearing
Early return to sport (without loss of fixation strength)

The choice of fixation devices for ACLR is mostly surgeon-dependent. Hakimi, et al. [25] found that in the UK the HS femoral fixation was done with a suspension device in 79% and interference screw in 18%. Of those using a suspension device, the EndoButton® was most common (48%), followed by TransFix® (26%) and RigidFix® (19%) (DePuy Synthes, Warsaw, Indiana). Tibial fixation was most commonly achieved by interference screw (57%) followed by Intrafix (30%) (DePuy Synthes, Warsaw, Indiana). With BPTB graft, the most popular femoral fixation was with an interference screw (66%) followed by suspension (34%). All surgeons used an interference screw for tibial fixation. Mahnik, et al. [26] in their study found that routinely used femoral fixation methods are suspensory fixation (62%), followed by cross pins (33%) and biodegradable interference screw (5%). A bioabsorbable interference screw was used in 97% of cases as a fixation method for tibial side fixation. Kim, et al. [27] concluded that the type of graft or fixation device did not affect the clinical outcome and stability.

**Figure 3:**
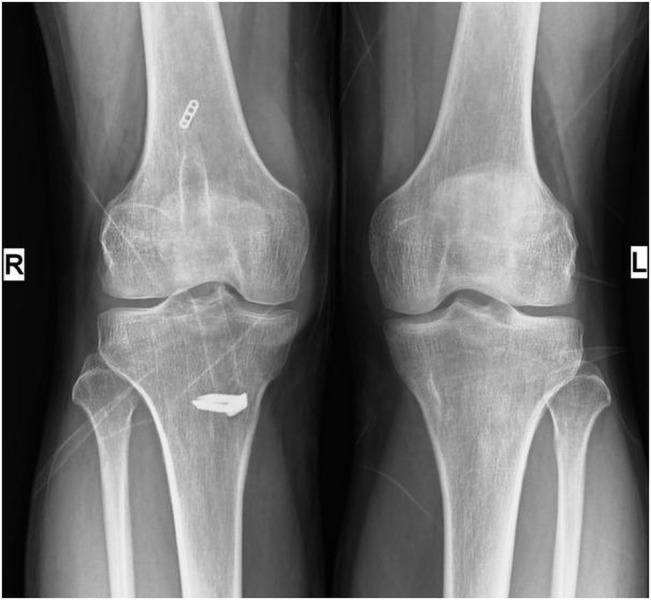
AP radiographs of the knees showing tunnel widening in the right femur and tibia after ACLR

We are of the opinion that the surgeons should select an ideal fixation device for ACLR depending upon patient’s condition and surgeon’s experience. Interference screws, either bioabsorbable or metal, are frequently used for the fixation of the bone plug of the BPTB graft into the femoral tunnel while, for the fixation of the hamstring graft, EndoButtons are commonly used. EndoButtons are mainly associated with high failure load and tunnel widening due to graft-related micro motions into the bony tunnel (Figure 3). Cross biodegradable pins and retro screws also can be used for graft fixation. The majority of the graft fixation implants are strong enough to hold the graft in place and selection of graft fixation device is done considering bone quality and graft type [27]

The choice of interference screw material has seen a recent change in the use pattern, from metal screws to biodegradable screws. However, Ma, et al. [28] found no difference between metal and biodegradable screw usage clinically. Noticeable tunnel widening was seen in both groups, especially on the femoral side. They also found that MRI studies at two to four years postoperatively showed no degradation of the bioabsorbable screw. Kong, et al. [29] found that a hamstring autograft fixed with EndoButton® on the femoral side had a good outcome compared to cross pin fixation at four-year follow-up. Tunnel widening developed in both groups, but this did not lead to surgery failure. They concluded that both cross pin and EndoButton® were useful materials for femoral tunnel fixation in hamstring ACLR surgery.

There are few newer devices available for ACLR, which would need further research and follow-up. The Endo Tunnel Device® (ETD) (ProInd, Cotia, São Paulo, Brazil, ) is a new cortical suspension device for femoral fixation described by Guglielmetti, et al. [30]. They found ETD to be a safe femoral fixation device; its use in both the trans-tibial and transportal techniques is technically simple and is associated with few intra- or postoperative complications. The advantages and disadvantages of this device are merely theoretical because there are no studies that compare it with other fixation devices. Calas, et al. [31] described the Cage For One (CFO) (Sacimex, Aix-en-Provence, France) system in which a looped four-strand semitendinosus tendon graft is used, leaving the gracilis tendon intact. The soft tissue graft is indirectly fixed into both the femoral and tibial tunnels by polyetheretherketone (PEEK) cages by use of polyethylene terephthalate tape strips. As the gracilis tendon is preserved, it retains flexion strength and rotatory stability of the knee. They concluded that with this method immediate stable fixation is achieved and brace-less rehabilitation can be started in the early postoperative period.

We believe that the method of choice for graft fixation is EndoButton®, followed by TransFix®, for the femoral tunnel and interference screw on the tibial side. The method preferred for the fixation of a bone plug in ACL reconstruction is an interference screw. The new methods of fixation, like ETD and CFO, need further research to advocate their routine usage.

### Transtibial vs transportal femoral tunnel

In ACLR surgery, the femoral tunnel is prepared either through a trans-tibial route or transportal, i.e., through the anteromedial arthroscopic portal. In the trans-tibial technique, the soft tissue graft is usually placed relatively in a vertical position while the transportal technique gives the surgeon a high freedom of selecting femoral tunnel position. Yau, et al. [32] found in their study that the use of a transportal technique in a single bundle ACLR provided improved positions in both the tibial and femoral tunnels as compared with the trans-tibial technique. Mandal, et al. [33] also found that patients operated with the transportal technique have good clinical knee scoring, less anteroposterior (AP) instability, and fast recovery.

Franceschi, et al. [34] reported ACLR, using a femoral tunnel drilled through an AMP, provided better rotational stability and anterior translation than drilling the femoral tunnel using the TT technique. This difference likely is not relevant from a clinical and functional viewpoint. However, there was no significant difference in the development of degenerative changes among these two groups radiologically at a minimum follow-up of five years. Chechik, et al. [35] noted that 68% of surgeons prefer the transportal technique while 31% prefer trans-tibial, and 1% prefer open method for femoral tunnel drilling worldwide. Thus, the transportal technique of femoral tunnel drilling seems to be a preferred method over trans-tibial technique as it gives better positioning of the femoral tunnel and good postoperative AP as well as rotational stability.

### Operative technique

Double bundle ACLR has been claimed by Stefani, et al. [36] to restore the anatomy of the torn ACL better than the single bundle ACLR. Double bundle reconstruction includes construction of both anteromedial and posterolateral bundles of ACL while in single bundle reconstruction only anteromedial bundle is addressed.

Kim, et al. found that, in the last ten years, the trend of ACLR has shifted towards anatomical reconstruction regardless of single or double bundle technique, and neither technique (i.e., either single bundle or double bundle reconstruction) has better clinical outcomes than the other.

Stefani, et al. [36] also found no significant difference between the two groups. Furthermore, it was observed that double bundle reconstruction is more time-consuming, costlier, and is associated with more complications (double trouble?), especially in cases of revision surgery. Still, the single bundle ACLR seems to be a preferred mode of reconstruction. A survey by Chechik, et al. showed that two-thirds of surgeons were performing single-bundle graft reconstructions compared to one-third who used the double bundle technique. Joshy, et al. [37] reported that the double bundle ACLR provides greater AP and rotational stability and significant improvement in the International Knee Documentation Committee (IKDC) score than the single bundle reconstruction.

By using two separate grafts, the double bundle ACLR seems to have the advantage of reconstructing both the anteromedial and posterolateral bundles, providing good stability throughout the entire range of motion, specifically, rotational stability. However, the caveat of double bundle reconstructions includes complicated surgery, costlier implants, and longer operative time. While the double bundle ACLR is apparently a more scientific procedure to create the natural anatomy of the ACL, single bundle ACLR procedures are still being done in the majority of medical centers. The single bundle ACLR has also seen a shift from using a trans-tibial to transportal technique for creating a femoral tunnel in a more anatomical and isometric zone of attachment of the ACL.

### Healing augmentation

Injured ACL does not heal because synovial fluid in the joint dissolves fibrin-platelet clots and prevents bridging. Fibrin platelet clot is a fibrin matrix in which platelet cytokines, growth factors, and cells are trapped, which helps to improve tissue healing. Thus, healing can be enhanced with the use of PRP, i.e. platelet rich as it has numerous growth factors that have shown their efficacy in healing. The benefits of PRP applied in ACLR have still not been demonstrated.

In a prospective study of 40 patients, Silva, et al. [38] in their radiological (MRI) follow-up at three months postoperatively did not notice any significant difference amongst different groups with the use of PRP compared to the non-PRP group. Nin, et al. [39] in their study also did not see any significant difference in the clinical or inflammatory parameters by the addition of PRP containing platelet-derived growth factors (PDGF) in primary ACLR with BPTB allograft cases at the two-year follow-up.

Mirzatolooei, et al. [40] studied the effect of PRP to prevent tunnel widening in ACLR and found that there was slightly less tunnel widening in PRP group, however, there was no noticeable difference in the clinical outcome of PRP versus non-PRP groups. Radice, et al. [41] found that with the use of PRPG (PRP gel), a complete homogeneity of the ACL graft can be seen earlier on MRI (in 179 days) compared to the non-PRPG group (in 369 days).Thus, they concluded that there was a reduction of the timing of 48% with the use of PRPG in the incorporation of the graft.

So far, most published studies have not been able to show any significant role of PRP in the acceleration of healing of soft tissue graft in bone tunnel in ACLR, while only a few studies have shown its beneficial role in the acceleration of healing.

### Rehabilitation

There is a vital role of rehabilitation after ACLR in achieving good functional outcomes. Rehabilitation also helps these patients to return to their pre-injury status earlier.

Kruse, et al. [42] and Saka [45] did not find postoperative bracing necessary or useful after ACLR. Active muscle strengthening exercises and ROM exercises were thought to be helpful and necessary for early recovery. Neuromuscular electric stimulation may also help in preventing quadriceps wasting postoperatively. Kruse, et al. and Beynnon, et al. [42-43] experienced no harmful effect of accelerated rehabilitation post-ACLR and found these patients to have similar outcomes as in non-accelerated rehabilitation at two years postoperatively.

In a study by Fukuda, et al. [44], they reported that an early start open kinetic chain (EOKC) exercise group showed a faster recovery in quadriceps strength than a late start open kinetic chain (LOKC) exercise group.

Cvjetkovic, et al. [46], in their prospective study of 70 males, examined the effect of a rehabilitation program on thigh muscle circumference and modified Tegner Lysholm score. Patients were divided into two groups each of 35 patients. Group A underwent “aggressive” or “accelerated” postoperative rehabilitation program while group B did not undergo the recommended rehabilitation protocol. Thigh muscle circumference and Lysholm scoring were evaluated preoperatively and postoperatively at one, three, six, and 12 months. The authors found a significant difference in thigh muscle circumference amongst the two groups and concluded that patients of Group A have better and faster functional recovery than Group B.

The primary goal of the ACLR rehabilitation program is to return to the sports activities by the sixth month. Recent rehabilitation programs are focused on reducing this time span with good functional outcome. With the above reviews, we conclude that a postoperative brace has no advantage. ”Accelerated” or “aggressive” rehabilitation protocol has a positive effect on thigh muscle strength and postoperative functional outcome in the ACL reconstructed patient.

### ACL reconstruction and osteoarthritis

In their randomized controlled study, Barenius, et al. [47] found that the prevalence of osteoarthritis (OA) increased three-fold after ACLR knee compared with a normal contralateral knee. The medial compartment was more frequently involved in OA. They also found no difference in the prevalence of OA between the BPTB graft and the quadrupled semitendinosus tendon graft.

Leiter, et al. [48] have found that the patients who underwent ACLR more than 12 years ago had incidence and severity of OA higher in ACL reconstructed knees than non-reconstructed knees. They also suggested that medial meniscus surgery has a strong prediction of developing OA. On the contrary, Ajuied, et al. [49] in their meta-analysis concluded that ACL injury increases the risk of OA, although ACLR reconstruction reduces the likelihood of the development of degenerative changes. They presumed that returning to sports activities after ACLR may exacerbate the development of OA. Struewer, et al [50] studied follow-up of 52 patients on an average of 10.2 years and found that, although patients had good surgical results and satisfaction at two-year and long-term follow-up, the prevalence of OA is high and developed in 25% of their patients. Li, et al. [51] also reported that 39% of their patients had radiographic evidence of OA at an average of 7.8 years postoperatively and concluded that patients who had undergone ACLR are at high risk of developing osteoarthritis compared with the general population. They further observed that obesity and Grade 2 or more chondrolysis in the medial compartment are the strongest predictors of the development of osteoarthritis after ACLR [52].

The majority of evidence available so far explicitly states that the risk of OA increases in patients after an ACLR. We believe that strongest predictor of the development of OA after ACLR are associated meniscal and osteochondral injury, obesity, return to aggressive sports, and the non-anatomical placement of the ACL graft.

## Conclusions

ACL reconstruction surgery has evolved considerably over the past few decades. Early reconstruction should be followed by accelerated rehabilitation, and delayed reconstruction is associated with poor outcome. Autograft yielded better results than allograft. BPTB-R was associated with better postoperative knee stability but with a higher rate of morbidity. However, in terms of functional outcome, both BPTB and hamstring graft were similar in the long-term. Most of modern fixation devices have enough strength to fix the graft in ACL reconstruction regardless of graft materials. All fixation devices have their distinct advantages and disad­vantages. Therefore, the choice of a fixation device should be based on the type of graft or quality of bone. Since there is a variety of options available today, selection of an optimum combination of the graft as well as fixation devices should be individualized to the patient’s condition and the experience of the surgeon. The double-bundle ACL reconstruction technique showed better outcomes in rotational laxity, although functional recovery was similar between single-bundle and double-bundle. The majority of patients were able to return to some sports participation after a rehabilitation protocol; however, almost a third of patients presenting with radiological signs of osteoarthritis with a minimum ten-year follow-up. Further advances in surgical techniques should continue to be developed so as to restore near-normal knee kinematics and anatomy. It would be preferable to apply patient-tailored rehabilitation protocols and return-to-sports criteria, based on individual characteristics.
